# Gut-microbe derived TMAO and its association with more progressed forms of AF: Results from the AF-RISK study

**DOI:** 10.1016/j.ijcha.2021.100798

**Published:** 2021-05-24

**Authors:** B.O. Nguyen, L.M.G. Meems, M. van Faassen, H.J.G.M. Crijns, I.C. van Gelder, F. Kuipers, M. Rienstra

**Affiliations:** a Department of Cardiology, University Medical Center Groningen, University of Groningen, Groningen, the Netherlands; b Department of Laboratory Medicine, University Medical Center Groningen, University of Groningen, Groningen, the Netherlands; c Maastricht University Medical Center+ and Cardiovascular Research Institute Maastricht, Maastricht, the Netherlands; d Department of Pediatrics, University Medical Center Groningen, University of Groningen, Groningen, the Netherlands

**Keywords:** AF progression, TMAO, Atrial fibrillation, Gut microbiome

## Abstract

**Introduction:**

The importance of gut microbiome in cardiovascular disease has been increasingly recognized. Trimethylamine N-oxide (TMAO) is a gut microbe-derived metabolite that is associated with cardiovascular disease, including atrial fibrillation (AF). The role of TMAO in clinical AF progression however remains unknown.

**Methods and results:**

In this study we measured TMAO and its precursor (betaine, choline, and L- carnitine) levels in 78 patients using plasma samples from patients that participated in the AF-RISK study. 56 patients suffered from paroxysmal AF and 22 had a short history of persistent AF. TMAO levels were significantly higher in patients with persistent AF, as compared to those with paroxysmal AF (median [IQR] 5.65 [4.7–9.6] *m*/*z* versus 4.31 [3.2–6.2] *m*/*z*, p < 0.05), while precursor levels did not differ. In univariate analysis, we observed that for every unit increase in TMAO, the odds for having persistent AF increased with 0.44 [0.14–0.73], p < 0.01. Conclusion: These results suggest that higher levels of TMAO are associated with more progressed forms of AF. We therefore hypothesize that increased TMAO levels may reflect disease progression in humans. Larger studies are required to validate these preliminary findings.

**Trial Registration number:** Clinicaltrials.gov NCT01510210.

## 1 Introduction

Recent studies have shown that gut microbiome has an important role in cardiovascular diseases [1], [2]. Trimethylamine N-oxide (TMAO) is a exogenous, gut microbe-derived metabolite that has been associated with cardiovascular diseases, such as atherosclerosis and progression of heart failure (HF) [3], [4]. In experimental studies, TMAO increases instability of atrial electrophysiology [5], and increased TMAO levels are also linked to new onset of atrial fibrillation (AF) regardless of age [6]. The role of TMAO and its precursors (betaine, choline, and L-carnitine) in clinical AF progression however remains unknown.

## 2 Methods

In this study we evaluated the role of TMAO and its precursors (betaine, choline, and L-carnitine) on clinical AF progression using patients from *Identification of a risk profile to guide atrial fibrillation therapy (AF-RISK)* study (NCT01510210). Detailed study design and outcomes have been previously described [7]. In short, the AF-RISK study was a multicenter, prospective, observational study, including patients with paroxysmal AF (total AF history < 2 years, or total AF history < 3 years in case of ≤ 2 AF episodes of ≤ 48 h per month terminating spontaneously) or with a short history of persistent AF (total AF history < 2 years, and total persistent AF duration > 7 days and < 1 year) in whom a rhythm control strategy was preferred [7]. The AF-RISK study was performed in compliance of the Declaration of Helsinki. The institutional review board approved study protocol and all patients gave written informed consent. After inclusion, all patients underwent baseline assessment including peripheral venous blood sampling for biomarker analyses (n = 499). Blood samples were processed and EDTA-plasma samples were stored at −80C. We randomly selected 78 non-fasted baseline blood samples from the total study population and used them to measure TMAO, betaine, choline, and L-carnitine. TMAO, betaine, choline, and L-carnitine in plasma were analyzed by ultra-high performance liquid chromatography in combination with isotope dilution tandem mass spectrometry (UPLC-MS/MS) as described in more detail previously [8]. Mass spectrometric detection was performed on a XEVO TQ-s system (Waters). Analytes were detected in positive mode and selected reaction monitoring mode. The respective quantifier ion transitions were as follows: *m*/*z* 76.15 > 58.3 for TMAO, *m*/*z* 118.2 > 59.3 for betaine, *m*/*z* 104.2 > 60.3 for choline, and *m*/*z* 162.2 > 103.25 for L-carnitine. All analytes were baseline separated from each other.

## 3 Statistical methods section

Continuous variables were presented as mean and standard deviation (SD) or, if not normally distributed, as median (interquartile range). Numbers were presented as counts (percentage). Differences between groups were analysed using student-*t-*test if normally distributed, or Mann-Whitney U if non-normally distributed. Linear regression was performed in a univariate manner. A p-value < 0.05 was considered statistically significant. Statistical analyses were performed using R package (Version 3.1.3; R Foundation for Statistical Computing, Vienna, Austria) or STATA (Version 14.2; StataCorp LLC; Tx, USA).

## 4 Results

**Fig. 1:**
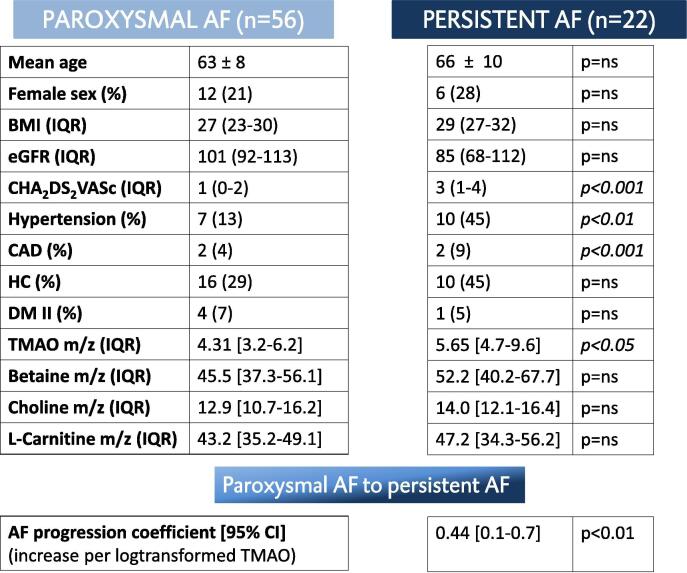
Schematic overview of AF burden in patients from AF-RISK with baseline characteristics of all participants including plasma TMAO levels (*m*/*z*), plasma betaine levels (*m*/*z*), plasma choline levels (*m*/*z*) and plasma L- carnitine levels (*m*/*z*), including an overview of the association between AF progression and the association with plasma TMAO levels (*m*/*z*). BMI = body mass index; IQR = interquartile range; eGFR = estimated glomerular filtration rate (mL/min/1.73 m2); CAD = coronary artery disease; HC = hypercholesterolemia; DM II = diabetes mellitus type II; TMAO = Trimethylamine N-oxide.

**Fig. 2:**
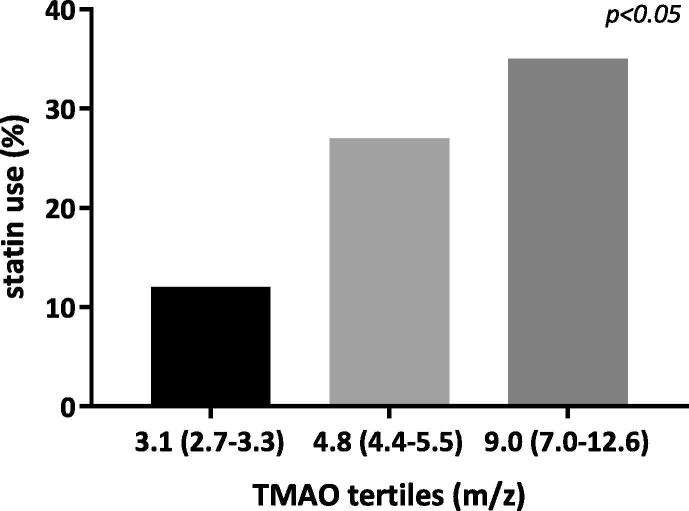
Statin use (percentage) and the effect on plasma TMAO levels per tertile.

In this AF-RISK substudy, mean age (±SD) of the study population was 63 ± 9 years, 77% were men, and 50% were diagnosed with HF with preserved ejection fraction or HF with reduced ejection fraction. TMAO levels were measured in a total of 78 patients in this randomly selected subgroup, 56 patients suffered from paroxysmal AF and 22 had a short history of persistent AF (Fig. 1). Patients with persistent AF had higher CHA_2_DS_2_-VASc scores (paroxysmal AF, median (IQR): 1 (0–2) versus persistent AF: 3 (1–4), p < 0.001), and more often had a history of hypertension (paroxysmal AF, n (%): 7 (13%) versus persistent AF: 10 (45%), p < 0.01), HF (paroxysmal AF: 8 (14%) versus persistent AF: 10 (45%), p < 0.05) and coronary artery disease (paroxysmal AF: 2 (4%) versus persistent AF: 7 (32%), p < 0.001). Patients with persistent AF more often used beta-blockers (n (%): 20 (91%) versus paroxysmal AF: 34 (61%) p < 0.05); digoxin (n (%): 6 (27%) versus paroxysmal AF: 3 (5%), p < 0.05); ACE-inhibitors (n (%): 12 (55%) versus paroxysmal AF: 12 (21%), p < 0.05); diuretics (n (%): 15 (68%) versus paroxysmal AF: 4 (7%), p < 0.05); new oral anticoagulants (n (%): 21 (95%) versus paroxysmal AF: 30 (54%) p < 0.001 and statins (n (%): 9 (41%) versus paroxysmal AF: 10 (10%), p < 0.05). No differences were seen in class I and III anti-arrhythmic use or ablation treatment between the persistent AF and paroxysmal AF patients. All other baseline characteristics and drugs were comparable between two groups. TMAO levels were significantly lower in those with paroxysmal AF, as compared to those with persistent AF (median [IQR] 4.31 [3.2–6.2] *m*/*z* versus 5.65 [4.7–9.6], p < 0.05 (Fig. 1). Precursor levels were similar in both paroxysmal and persistent AF: median [IQR] betaine 45.5 [37.3–56.1] versus 52.2 [40.2–67.7], p = ns; choline 12.9 [10.7–16.2] *m*/*z* versus 14.0 [12.1–16.4], p = ns; L-carnitine 43.2 [35.2–49.1] versus 47.2 [34.3–56.2], p = ns, respectively (Fig. 1). For every unit increase in TMAO, the odds for having persistent AF increased with 0.44 [0.14–0.73], p < 0.01 (Fig. 1). The increased use of statins was not associated with a difference in levels of TMAO, betaine, choline or L-carnitine (p = ns for all) (Fig. 2).

## 5 Discussion

In this small explorative and hypothesis-generating study, we observed that in the AF-RISK study TMAO levels were higher in those with persistent AF as compared to patients with paroxysmal AF. However, this was not the case for its precursor levels. This suggest that an association between TMAO levels and AF-progression may exist, and that future, larger and adequately powered studies are required to evaluate this hypothesis. Of note, TMAO levels were already shown to improve risk net classification for new-onset AF, regardless of traditional AF risk factors or dietary choline intake [6]. They may furthermore reflect increased electrophysiological atrial instability and enhanced atrial inflammation, as TMAO injections into 4 major atrial ganglionated plexi in experimental studies enhanced electrophysiological atrial instability and, when using a model of rapid atrial pacing, induced increased acute electrical remodeling including upregulation of inflammatory cytokines that belong to the p65 NF-κB signaling pathway [5]. Larger studies will be essential to validate and bolster our preliminary findings if TMAO levels may also reflect AF progression.

## 6 Study limitations

In this study, patients with higher TMAO levels more often reported use of a statin. Although it is not entirely clear how statins influence gut microbioma, a very recent study demonstrated that treatment with rosuvastatin inhibited TMAO-precursor metabolisation, especially in those patients with a more favorable response to rosuvastatin treatment. These results suggest that although statins may not alter gut microbioma composition, they may influence gut microbioma function, especially in those patients with a favorable treatment response [9]. We, however, were not able to establish an association between statin use and levels of TMAO or its precursors. The association between TMAO levels and more progressed forms of AF may therefore not be straightforward, especially not in those patients who also suffer from HF, and larger studies will be needed to evaluate if TMAO levels have predictive value in AF progression and AF management.

This study is a cross-sectional study and results are thus descriptive and do not reflect causality of TMAO and AF progression. Furthermore, TMAO levels were analyzed in non-fasted blood samples and diet of each patient was unknown. The sample size of this study was very small, and therefore study observations are explorative and hypothesis-generating and cannot be used to draw robust conclusions. Additionally, because of the limited sample-size more elaborate and extensive statistical testing (such as multivariate regression analyses or propensity score matching) are not feasible. Of note, AF-RISK was designed to evaluate AF-progression in patients with AF and did not include a participants without AF; we could therefore not compare TMAO levels in patients with and without AF.

## 7 Conclusion

In conclusion, in this small explorative and hypothesis-generating study we show that increasing TMAO levels are associated with more progressed forms of AF, while this is not the case for its precursor levels betaine, choline and L-carnitine. We hypothesize that increased TMAO levels may reflect AF disease progression in humans. Larger studies are essential to validate and bolster our findings, especially since the association between TMAO levels and AF progression may not be straightforward as it may be influenced by other factors such as statin use.

## Funding

This work was supported by an unrestricted grant from the Noaber Foundation paid to the University Medical Center Groningen. AF-RISK was supported by the Netherlands Heart Foundation (NHS2010B233). We acknowledge the support from the Netherlands Cardiovascular Research Initiative: an initiative with support of the Dutch Heart Foundation, CVON 2014–9: Reappraisal of Atrial Fibrillation: interaction between hyperCoagulability, Electrical remodelling, and Vascular destabilisation in the progression of AF (RACE V).

## Declaration of Competing Interest

The authors report no relationships that could be construed as a conflict of interest.
